# Calprotectin, an Emerging Biomarker of Interest in COVID-19: A Systematic Review and Meta-Analysis

**DOI:** 10.3390/jcm10040775

**Published:** 2021-02-15

**Authors:** Raphael Udeh, Shailesh Advani, Luis García de Guadiana Romualdo, Xenia Dolja-Gore

**Affiliations:** 1 Hunter Medical Research Institute Building, School of Medicine & Public Health, The University of Newcastle, Lot 1, Kookaburra Circuit, New Lambton Heights, NSW 2305, Australia; xenia.doljagore@newcastle.edu.au; 2 Terasaki Institute of Biomedical Innovation, Los Angeles, CA 90024, USA; shailesh.advani735@gmail.com; 3 Hospital Universitario Santa Lucía, Calle Minarete s/n, 30202 Cartagena Murcia, Spain; guadianarom@yahoo.es

**Keywords:** COVID-19, calprotectin, biomarker, systematic review, meta-analysis

## Abstract

COVID-19 has been shown to present with varied clinical course, necessitating a need for more specific diagnostic tools that could identify severe cases and predict outcomes during COVID-19 infection. Recent evidence has shown an expanded potential role for calprotectin, both as a diagnostic tool and also as a tool in stratifying COVID-19 patients in terms of severity. Therefore, this systematic review and meta-analysis aims to evaluate the levels of calprotectin in severe and non-severe COVID-19 and also identify the implication of raised calprotectin levels. MEDLINE, EMBASE, The Cochrane Library, Web of science and MedRxiv were searched. Meta-analysis was done to compare the serum/fecal levels of calprotectin between severe and non-severe COVID-19 infections. A total of ten studies included in the review (eight had quantitative data while two were qualitative). A pooled analysis of the eight studies from 613 patients who were RT-PCR positive for COVID-19 (average age = 55 years; 52% males) showed an overall estimate as 1.34 (95%CI: 0.77, 1.91). In conclusion, calprotectin levels have been demonstrated to be significantly elevated in COVID-19 patients who develop the severe form of the disease, and it also has prognostic importance.

## 1. Introduction

Wuhan city in Hubei province of China, in December 2019, became the epicenter of an epidemic that turned into a pandemic within three months after spreading to 114 countries [1,2]. The global burden of the disease today (6 February 2021) is about 104 million cases and over 2 million deaths [2]. Initially called 2019-nCoV and later severe acute respiratory syndrome coronavirus 2 (SARS-CoV-2), it was branded COVID-19 by WHO in February 2020 [1]. COVID-19 is a highly infectious viral disease that affects the lungs and immune system, clinically presenting with fever, cough and dyspnea [3]. Varied clinical course has been reported for COVID-19 infection—some mild forms that recover, while others worsen to severe forms characterized by defective oxygenation that presents with a multi-organ dysfunction and death [3,4]. COVID-19 has been demonstrated to be a highly inflammatory disease riddled with abnormally controlled immune response [4,5,6]. Despite huge advances, the molecular mechanism underpinning the pathophysiology is still largely unknown [7]. Unfortunately, the global public health burden of the pandemic has continued to worsen with increasing morbidity and mortality rates [2,5]. Numerous molecules have been assessed to serve as severity marker for COVID-19, but there is still not one that is universally recognized.

Calprotectin (aka S100A8/A9, calgranulin A and B, alarmins, CLP) is a 36.5 small calcium and zinc-binding heterodimer, derived from neutrophils and macrophages that often associates with its main receptor, TLR4 (Toll-like receptor 4) via a non-covalent bond to mediate downstream signaling with active involvement in inflammation [8,9]. S100A8/A9 are fundamentally expressed and released by neutrophils and macrophages such that they make to 45% of all cytosolic proteins in neutrophils [8]. As an acute phase reactant, its expression level is often increased following infection, trauma and in inflammatory diseases [8,10,11], during which the neutrophils and other cells are induced to release cytokines, reactive oxygen species (ROS) and nitric oxide (NO). Its stability at room temperature makes it a potential biomarker in inflammatory diseases including COVID-19 [8,12]. Its concentration in several body fluids (stool, serum, synovial, salivary, etc.) have been reported, with serum levels often below 1microgram per ml in normal individuals and rising by 100-folds following inflammation [8,10]. The half-life of calprotectin has been documented to be 5 h and unlike other acute phase reactants which are produced in the liver—this heterodimer is synthesized and secreted locally at the inflammatory site [8]. Postulated functions for calprotectin include intracellular role in cell migration, cell maturation, arachidonic acid metabolism; extracellularly, in binding to TLR4 as well as RAGE (receptor for advanced glycation end products), thus activating the inflammatory signal pathways. Its role in the control of cell chemotaxis is known [8,9,10]. Its predictive role in the outcome of some inflammatory diseases including rheumatoid arthritis and inflammatory bowel disease has been reported [8,10,13].

Zhou et al. had earlier reported the raised markers of cytokine storm (Interleukin-6, IL-6; Interleukin-10, IL-10; Tumor necrosis factor-α, TNF-α) in COVID-19, and also described IL-6 as the best predictor of death in their case control study on COVID-19 [1]. Recent studies state that calprotectin level is raised in severe COVID-19 and thus could discriminate the various forms of COVID-19 as well as predict the outcome in terms of who ends up in the intensive care unit (ICU) or would die [12,14]. Recent studies suggest that several biomarkers including lymphopenia, and raised C-reactive protein (CRP), procalcitonin (PCT), D-dimer, creatine kinase (CK), aspartate aminotransferase (AST), alanine transaminase, creatinine and serum amyloid A are significantly associated with poor prognosis in COVID-19 patients [15,16,17]. However, there has been growing evidence that calprotectin is a better biomarker than the aforementioned markers [8,12,14,18]. The purpose of this review is to carefully evaluate the levels of calprotectin (serum, plasma or fecal) in severe and non-severe COVID-19, and also identify the implications of raised levels of calprotectin. Meta-analysis was employed to subdue the challenges of underpowered individual studies, so as to enhance accuracy, as well as assess its correlation with some important clinical parameters including demographic data. This data will provide physicians with a tool to stratify COVID-19 patients in terms of severity to allow for early intervention, as well as help to predict outcome including risk of death.

## 2. Materials and Methods

### 2.1. Data Source and Search Strategy

This study is a systematic review, and a literature search was conducted to identify recent papers on this subject. MEDLINE, EMBASE, The Cochrane library [Cochrane Database of Systematic Reviews, Cochrane Central Register of Controlled Trials (CENTRAL), Cochrane Methodology Register, Web of Science and MedRxiv (for preprints) were searched using the following keywords: “COVID-19”, “Novel coronavirus”, “calprotectin”, “S100A8/A9” from 1 December 2019 to 5 December 2020. The review followed the PRISMA (Preferred Reporting Items for Systematic Reviews and Meta-analysis) guidelines [19].

Titles and abstracts were independently screened using the selection criteria to determine the eligible studies. Then, full texts of the studies were closely assessed and studies were either included or excluded accordingly. Consensus discussion and agreement were reached for any paper that was contentious in its content to clear any ambiguity. Manual search was finally conducted in the reference list of included studies for any article missed out during the electronic search.

### 2.2. Eligibility Criteria and Data Extraction

Essentially, adults ≥ 18 years that tested positive (by RT-PCR) to COVID-19 virus, as well as studies that assessed calprotectin level are included. Further, studies that compared and reported data on the severe and non-severe forms of COVID-19 were included. Severe COVID-19 infection for this review describes distress due to poor respiratory function that needs hospital admission into the ICU and/or mechanical ventilation [12,20]. It also includes all critical cases of COVID-19 infection. On the other hand, non-severe COVID-19 infections include the mild and moderate forms, which could be on admission but not in the ICU. The outcome of interest is primarily the difference in the mean calprotectin levels of the severe and non-severe groups across the included studies, as well as its association with the various reported clinical and/or laboratory profiles. Observational studies were included while reviews, guidelines and irrelevant assay studies were excluded (Table 1). It is worth mentioning that relevant editorial letters that reported on some data from case series studies were included too. This decision was due to the low yield of the electronic search and paucity of relevant data on this novel field of COVID-19 research.

Data extraction was conducted first for study characteristics (author name, mean age, gender proportions, study design, peer review status, location, hospitalization, ICU need, deaths). Secondly, results are summarized in two tables (one for the quantitative studies and another for the qualitative studies).

### 2.3. Quality Assessment 

Each of the included studies were independently assessed by two authors for quality using either the Newcastle-Ottawa scale (NOS) or the National Institute of Health (NIH) quality assessment tool). The NOS was employed in assessing the case-control (retrospective) or the cohort studies. It comprises three main parameters: selection, comparability and exposure (for case control studies)/outcome (for cohort studies). The case series studies, on the other hand, were assessed using the nine-parameter NIH tool. Any disagreement between the review authors with regards to the risk of bias in any of the studies was settled by discussion. The rating and overall score for each study are presented in a table. Each tool scores a study as either low quality (<5), moderate quality (5–7) or good quality (>7).

### 2.4. Statistical Analysis

A narrative synthesis of the results of each of the included studies is outlined. Another extraction table outlines summaries of the findings of each study by estimating their standardized mean difference (SMD) for continuous outcomes or odds ratios (for binary outcomes), using the data given in the published articles or as obtained from authors of those studies. Studies were categorized based on whether serum calprotectin or fecal calprotectin was the outcome measure estimated and analyzed as thus. To evaluate the difference in the mean values of the serum/fecal calprotectin in the severe and control groups of COVID-19 in each study, we employed Stata/IC version 16.1 and Microsoft excel to pool the estimates of these studies via a meta-analysis. SMD for each study was estimated, 95% confidence interval computed with the *p*-values and forest plot produced for homogenous studies.

A pre-specified alpha of 5% was employed for all tests, and heterogeneity was assessed using *I*_2_ statistic where values > 50% were considered significant between group heterogeneity—in such instance, a random-effects model (REM) was used. Otherwise, a fixed-effect model (FEM) was used [21]. *I*_2_ is the chi-squared statistic that describes the percentage of the variability in the pooled estimate that is due to heterogeneity and not by probability [21,22]. It is often employed to test for between-study heterogeneity in meta-analysis. *I*_2_ values less than 50% are often considered to show heterogeneity that is not statistically significant, while values more than 50% are considered to show substantial heterogeneity which is statistically significant [21]. Potential sources of heterogeneity were sought using subgroup analysis and meta-regression analysis. Any publication bias in the selected studies was assessed using funnel plot (plot of effect sizes against the inverse of standard errors) where asymmetry suggests a publication bias. Begg’s rank test describes a correlation of effect sizes to their respective variances and a strong correlation implies significant bias (often used for binary outcomes). Egger’s test was also used, which is employed for continuous outcomes and produces a weighted regression of effect size on their precision [23,24]. Publication bias is considered to be present if Egger’s test produces a *p*-value < 0.1 [24].

## 3. Results

### 3.1. Literature Search

**Figure 1:**
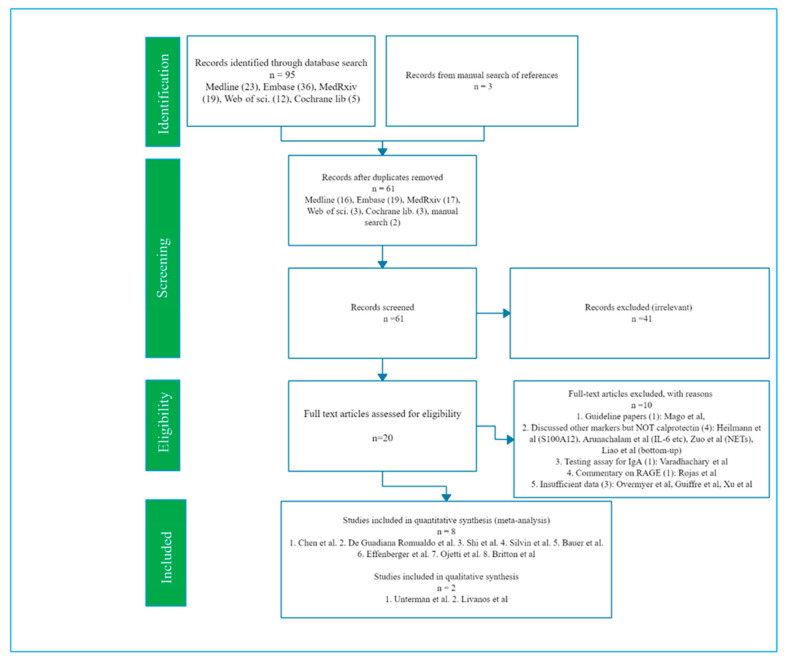
PRISMA 2009 Flow Diagram [19].

The initial search yielded a total of 95 published records from MedLine, EMBASE, MedRxiv, Web of science and Cochrane Library (Figure 1). Three papers were found through manual search of the references of included studies, making it 98 records in total. Following the removal of 37 duplicate papers, the record came down to 61. On a close examination of the titles/abstracts, 41 papers were found to be unrelated to calprotectin or COVID-19 and were thus excluded for their irrelevance to the index review. Thus, 20 articles were left for further assessment. The full text of these 20 studies were reviewed, and 10 articles that did not have enough data or was not relevant were excluded (Figure 1). Finally, 10 studies were included in the review as they were related to Calprotectin in COVID-19 (eight quantitative studies and two qualitative studies) (Figure 1; Table 2 and Table 3).

### 3.2. Study Characteristics

Ten studies were included overall: eight quantitative and two qualitative (Figure 1; Table 2). Among the eight included quantitative studies, five studies discussed the comparison of serum levels of calprotectin in severe and non-severe COVID-19 patients [12,14,25,26,27] (Table 3). Three of the studies reported a comparison of the fecal calprotectin [18,28,29] (Table 3). The two included qualitative studies conducted an RNA-sequence analysis of a single cell [33,34] with reports documenting more elevated levels of calprotectin expression in severe compared to non-severe COVID-19 (Table 4). However, specific data on the exact levels were either unavailable or insufficient to be included in the pooled analysis.

**Figure 2:**
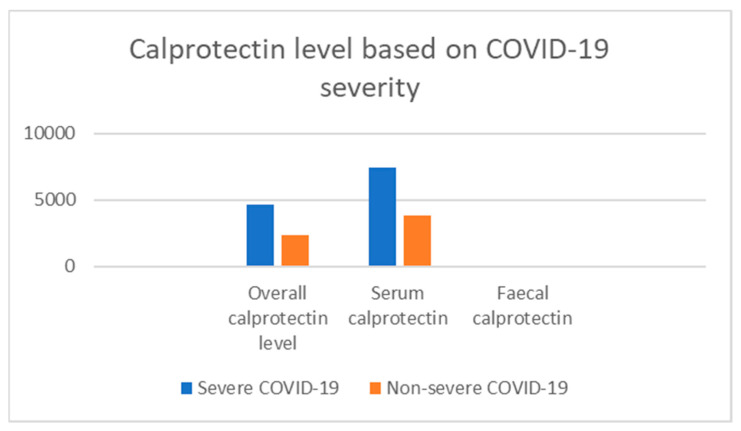
Bar chart comparing calprotectin levels in severe and non-severe COVID-19.

Eight studies with a total of 613 patients presented data that could be used for meta-analysis (Table 3). All 613 patients had a laboratory-confirmed COVID-19 infection. The mean serum/fecal calprotectin was compared between the severe and non-severe groups of COVID-19 infection in these included studies (Figure 2). Among the included studies, all of the serum quantitative studies were considered to be of good quality [12,14,25,26,27] while two of the fecal studies were of good quality [28,29] except Effenberger et al. [18], which was considered to be of moderate quality (Table 2). Further, the two included studies in the qualitative category had good (Unterman et al.) [33] and low (Livanos et al.) [34] quality respectively (Table 4). Review authors state that consensus discussion and agreement were reached with regards to rating of quality scales for each study while applying the NOS and NIH tools (Tables S1 and S2) [30,31,32].

This review also compared the mean calprotectin level for severe and non-severe COVID-19 for the combined calprotectin, serum calprotectin and fecal calprotectin results (Figure 2). The mean calprotectin levels for the severe and non-severe COVID-19 groups respectively are [4660, 2393] for combined, [7425, 3823] for serum calprotectin, and [51.3, 10.4] for fecal calprotectin levels (Figure 2). All the included quantitative studies reached statistical significance except for Britton et al. [29] and Bauer et al. [27] (*p*-value = 0.12 and 0.15) (Table 3).

### 3.3. Meta-Analysis, Forest Plot and Sensitivity Analysis

**Figure 3:**
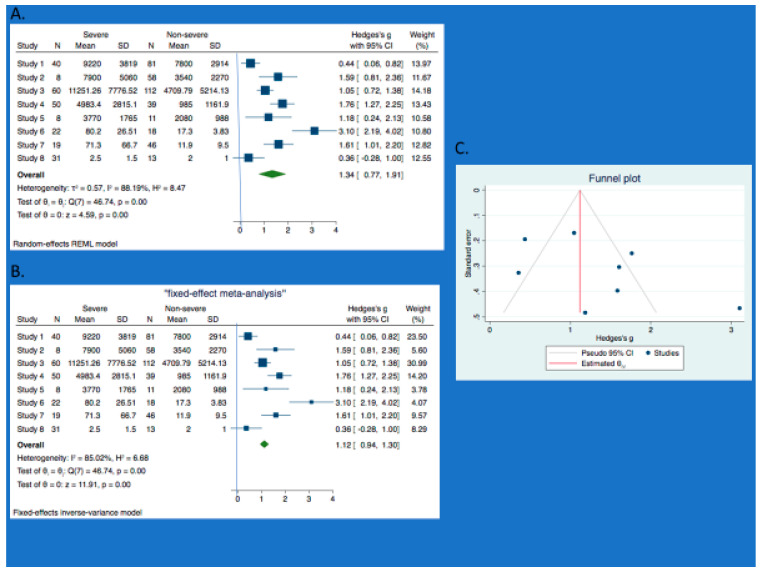
Meta-analysis (Total cohort). A. Forest plot (REM) and B. Forest plot (FEM) comparing the mean differences in calprotectin level between severe and non-severe COVID-19. Studies 1–8 are respectively—Chen et al [14]; De Guadiana et al [25]; Shi et al [26]; Silvin et al [12]; Bauer et al [27]; Effenberger et al [16]; Ojetti et al [28]; Britton et al [29]. C. Funnel plot showing some publication bias.

The forest plot of our meta-analysis showed the pooled overall estimate as 1.34 (95%CI: 0.77, 1.91) (Figure 3A,B). Across the eight studies involved in the pooled analysis, the heterogeneity (I^2^) was 88%. I^2^ > 50% is considered to show substantial heterogeneity [21,22]. Congruent upon the I^2^, is the chi-squared statistic (Q) which is 46.7 on seven degrees of freedom with a low *p*-value of 0.001, which is statistically significant.

The vertical line at 0 is the “line of no effect,” which means that the difference between calprotectin level for severe and non-severe COVID-19 is not statistically significant (Figure 3A,B). The horizontal lines through the square of each study represent the 95% confidence interval of their point estimates. Hence, statistical significance for a study is achieved if its 95% confidence interval does not cross 0, while any study whose horizontal lines crosses 0 (line of no effect) means there is no statistical significance.

According to Figure 3A,B, the forest plots of the pooled analysis show that the 95% confidence intervals of all the studies did not cross 0 except for one study (Britton et al. [29]). So, at study level, all the studies achieved statistical significance except for Britton et al. [29]. The overall estimate for the pooled analysis has a 95% confidence interval that did not cross 0. So, there is statistical significance at the pooled analysis level. Further, the 95% confidence interval of the overall estimate lies on the right of the line of no effect, which supports our hypothesis that calprotectin level is significantly higher in severe cases of COVID-19 compared to the non-severe forms (Figure 3A,B). The statistical significance of the overall estimate for the pooled analysis is further supported by the *p*-value of 0.001 (Figure 3A,B).

Due to the high heterogeneity in the pooled analysis in which we used the random effect model, a sensitivity analysis was done by attempting the fixed effect model. The fixed model produced a lesser overall estimate 1.12 (95% CI: 0.94, 1.30). Little effect on heterogeneity (I^2^ = 85%; Q did not change) (Figure 3A,B). Considering that the REM and FEM gave similar result, this entails that any small-study effect will be very insignificant [20]. However, further sensitivity analysis using the single study effect by excluding the three outliers in the plots (Chen et al. [14], Effenberger et al. [18] and Britton et al. [29]), showed the pooled estimate as 1.42 (95% CI: 1.07, 1.76) for REM and 1.34 (95% CI: 1.11, 1.57) for FEM (Figure S1A,B). There is also a 43% reduction in the heterogeneity report with huge decrease in Q, which is 7.1 on four degrees of freedom and *p*-value of 0.13 (Figure S1A,B).

### 3.4. Subgroup Analysis and Meta-Regression

Since there is substantial heterogeneity, there is a need to explore the data for possible causes. First of all, we conducted a subgroup analysis such that the data were analyzed as study group either reporting on serum calprotectin [study 1–5] [12,14,25,26,27] or reporting on fecal calprotectin [study 6–8] [18,28,29]. The results respectively are as follows 1.17 [0.66, 1.67] I^2^ = 79% *p*-value 0.001, and 1.66 [0.13, 3.19] I^2^ = 93% with a *p*-value = 0.03. Owing to the small number of included studies, a meta-regression may not be justifiable [22].

### 3.5. Publication Bias

Substantial heterogeneity was further explored for bias [22]. For the serum subgroup, there was no evidence of publication bias (Figure S1D) and Egger’s test (*p*-value = 0.45). The fecal subgroup had some evidence of publication bias as shown by funnel plot with some asymmetry (Figure S1E) and Egger’s test (*p*-value = 0.16). The total cohort of studies showed some evidence of publication bias, with funnel plot showing some asymmetry (Figure 3C) and Egger’s test of *p*-value = 0.13. Further, running the pooled analysis with all eight studies except three (Chen et al. [14], Effenberger et al. [18] and Britton et al. [29]), showed some improvement, with a resultant symmetrical funnel plot (Figure S1C) and improved Egger’s test (*p* = 0.62).

## 4. Discussion

Our meta-analysis addressed heterogeneity among the included studies. It is worth noting that the total cohort, serum cohort and fecal cohort of studies, all showed substantial heterogeneity. This high level of heterogeneity in these cohorts seems to be explained by three “outlier” studies: Chen et al. [14], Effenberger et al. [18] and Britton et al. [29]. Effenberger et al. is about the most obvious outlier study in the pooled analysis with the highest effect size [18]. It demonstrated that high fecal calprotectin level in COVID-19 signifies gut tropism, with levels significantly higher in COVID-19 patients having diarrhea [18]. Chen et al. demonstrated that raised serum calprotectin level correlated with need for oxygen support and overall poor outcome in COVID-19 patients [14]. Britton et al. study showed that fecal calprotectin did not correlate with gastrointestinal symptoms in COVID-19 patients [29]. However, this result was not statistically significant. Excluding these three studies reduced the heterogeneity from 88% to 45%.

### 4.1. Diagnostic Performance of Serum Calprotectin in Selecting Severe COVID-19

Shi et al. reported that plasma calprotectin among those hospitalized (*n* = 94) was able to select patients who needed mechanical ventilation (8039 +/− 7031 ng/mL, *n* = 32) as opposed to those who did not need intubation (3365 +/− 3146, *n* = 62, *p* < 0.0001) [26]. Silvin et al. also showed higher levels of serum calprotectin, classical monocytes and decreased levels of non-classical monocytes in the severe categories of COVID infection [12]. This report is also supported by the work of De Guadiana et al. [25] and Bauer et al. [27]. However, the work of Bauer et al. [27] did not reach statistical significance.

### 4.2. Prognostic Value of Serum/Fecal Calprotectin

A marked difference in the serum calprotectin levels between COVID-19 survivors and non-survivors has been demonstrated by de Guadiana et al. [25]. They further showed that serum calprotectin had a positive correlation with ferritin (*r* = 0.359; *p* = 0.003), CRP (*r* = 0.686, *p* = 0.001) and D-Dimer (*r* = 0.330, *p*= 0.007) [25]. Silvin et al. demonstrated that a reduced frequency of non-classical monocytes (using a flow cytometry assay) along with raised serum calprotectin level could indicate patients who will develop severe forms of COVID-19 [12]. Hence, serum calprotectin could have a predictive value in identifying high risk patients that would end up in the ICU.

Two clinical parameters have been identified to have a predictive value that correlated well with calprotectin levels viz diarrhea and comorbidities [12,18]. There is a growing evidence suggesting that diarrhea in COVID-19 has a clinical significance [35]. At a prevalence of 5–10%, the presence of GI symptoms (nausea, vomiting and diarrhea) has been linked to increased severity of respiratory symptoms, as indicated by more need for ventilator support or ICU admission [4,35,36]. However, a recent retrospective study of 44 COVID-19 patients stated that fecal calprotectin did not correlate with gastrointestinal (GI) symptoms [29]. This study nonetheless did not achieve statistical significance [29]. A different study, Ojetti et al. demonstrated a significant correlation between elevated fecal calprotectin and COVID-19 disease severity (often presenting as pneumonia ± systemic involvement like diarrhea, vomiting) [28]. However, they stated further that even in the absence of GI symptoms, such asymptomatic patients will still show high calprotectin levels [28]. Comorbidities have been shown to be an important predictor of severe COVID-19 [4,12]. The recent work of Silvin et al. had shown that although plasma calprotectin correlated well with COVID-19 severity and also with comorbidities; however, the correlation with severity far exceeds that of comorbidity [12]. The study found no association between calprotectin level and age [12].

To support the predictive capacity of calprotectin demonstrated by earlier studies, an exposition by Chen et al. showed that very high levels of calprotectin was associated with poor overall survival. This is supported by some recent works that showed elevated serum calprotectin correlates strongly with risk of death in COVID-19 often due to thrombotic issues [26,37]. When compared to COVID-GRAM risk score developed for predictive purposes [38], Chen et al. had hypothesized that calprotectin as a single predictive parameter has a superior predictive accuracy [14]. To demonstrate this, they compared the area under the curve (AUC) in its receiver operating characteristic curve (ROC) analysis of calprotectin, HMGB1, COVID-GRAM risk score and calprotectin/HMGB1 combination for predicting admission to ICU and potential death. The AUC results were respectively 0.860, 0.781, 0.810, 0.901 (for predicting ICU admission) and 0.875, 0.694, 0.818, 0.881 (for predicting potential death) [14].

### 4.3. Diagnostic Superiority of Calprotectin over Other Acute Phase Reactants

Significant elevation of acute phase reactants such as CRP, ferritin and D-dimer have been shown to be good predictors of poor survival in COVID-19 patients [39]. Evidence from the available data suggests that calprotectin might be diagnostically superior to IL-6, D-dimer and other markers of inflammation. Effenberger et al. reported a correlation between fecal calprotectin and serum IL-6 in COVID-19 in patients having diarrhea [18]. Mago et al. also showed that fecal calprotectin is more accurate and is therefore a superior marker compared to CRP, ESR in IBD patients with COVID-19 [40].

De Guadiana et al. also showed that calprotectin has a predictive capacity (using the AUC ROC assessment for deaths during hospital admission) similar to that of D-Dimer and CRP, but much better than that of ferritin [25]. This study adjusted for SOFA (Sequential organ failure assessment) score using IPW (inverse-probability weight) method. Similarly, Chen et al. demonstrated that calprotectin is more sensitive than HMGB1 in picking up mild/moderate forms of COVID-19 infection—as those admitted in the general ward had a significant elevation in calprotectin level but not in HMGB1 [14]. On the other hand, those in the ICU (severe COVID-19) had both calprotectin and HMGB1 raised. The study further stated that calprotectin has a predictive accuracy much better than COVID-GRAM score and other clinical/laboratory markers.

Furthermore, several studies demonstrated the predictive capacity of calprotectin in different prognostic scenarios: Shi et al. compared AUC for calprotectin on the need for invasive ventilation (calprotectin = 0.794, CRP = 0.614, ferritin = 0.562) [26]; Silvin et al. showed the ROC AUC of plasma calprotectin as a discriminating biomarker (calprotectin = 0.9590, non-classical monocytes = 0.8705, CD16low = 0.7983, IFNα = 0.5613) [12]. Further, Bauer et al. showed the ROC AUC of plasma calprotectin with regards to multiple organ failure within 72 h as (calprotectin = 0,87, lactate = 0.79, CRP = 0.70, 0.75) and ICU admission as (calprotectin = 0.70, lactate = 0.80, CRP = 0.66, PCT = 0.60) [27]. For the ICU admission, calprotectin performed better than CRP and PCT but not with lactate. The study acknowledged an intrinsic source of bias that favors lactate as hyperlactatemia is a commonly used biomarker for ICU admission [27].

As a biomarker for COVID-19, serum calprotectin is unique due to the following intrinsic properties: (1) it is easy to quantify; (2) its small size makes it a more sensitive and active marker—as it diffuses easily between the blood and tissue. Hence, it is the most abundant inflammatory marker seen in the plasma of patients with severe COVID-19; (3) its results are simple to interpret; (4) it has a strong correlation with qSOFA and oxygen demand; (5) it is powerful on its own either in selecting patients with severe COVID-19 or in predicting poor outcome. Hence, it is an important single parameter biomarker model (unlike COVID-GRAM scoring model that combines a number of clinical, laboratory and radiological parameters to reach a given score); (6) it has a much better AUC ROC than CRP, ferritin and LDH; (7) it has the capacity to indicate early COVID-19 patients who will either end up in the ICU or die [12,14,18,26]. Therefore, we hereby propose that calprotectin stands out as an indispensable biomarker for COVID-19.

### 4.4. Strength and Limitations

To our knowledge, this is the first comprehensive and extensive systematic review and meta-analysis to assess the prognostic value of calprotectin in distinguishing between COVID-19 severity in patients and its impact on clinical outcomes, since the outbreak over 12 months ago. The meta-analysis had a pooled population size of 613 patients (average age = 55 years; 52% males) who were RT-PCR positive for COVID-19. The overall pooled estimate presents with enhanced accuracy, a better picture of calprotectin’s diagnostic and prognostic significance. Further, despite being observational in nature, most of the studies were of good quality overall. Lastly, the included studies overlapped fairly well in terms of their description of severe and non-severe forms of COVID-19.

This review has the following limitations. Firstly, some of the individual studies of the meta-analysis were underpowered by small number of study participants. Secondly, huge variations in the populations and clinical attributes contributed to some substantial heterogeneity in the pooled estimate of the study. Further, the study is limited by existing variations in calprotectin (serum/fecal) normal range across different laboratories in these studies. Owing to the high heterogeneity, there is a need to be cautious in interpreting our result. Lastly, there is lack of adequate data since most of COVID-19 research is still in the infancy stage, and we continue to learn more every day on how the virus operates both within the human system and the environment. Such paucity of data warrants that we exercise some caution in generalizing our findings while we wait for more data. Hence, one cannot overemphasize the need for more data (both observational studies and clinical trials) on this subject which is relatively a novel field in COVID-19 research, as such will help to further expand our understanding of calprotectin in COVID-19 and strongly establish calprotectin’s role in COVID-19.

We identified a systematic review on the use of fecal calprotectin in COVID-19 that demonstrated that COVID-19 could lead to raised fecal calprotectin mostly in the presence of GI symptoms [41]. It included studies published up until 27 September 2020. However, the paper was focused on showing how challenging it is to differentiate COVID-19 infection from disease flares in inflammatory bowel disease. The review also did not have any direct studies of fecal calprotectin in IBD patients having COVID-19 infections. Furthermore, no meta-analysis was done.

COVID-19 continues to spread rapidly leading to massive rates of infection across the globe, with rising death rates most especially in the USA and across Europe. Given the rapidly evolving clinical understanding of COVID-19, it remains crucial to identify biomarkers that can help predict both severity and outcomes associated with COVID-19. Such will facilitate the formulation of efficient management guidelines, geared towards improved outcomes.

## 5. Conclusions

Our review and pooled analysis demonstrate that circulating calprotectin levels are elevated in COVID-19 infections. More importantly, these higher calprotectin levels could select severe COVID-19 from non-severe forms, predict poor outcome as well as predict gut inflammation in COVID-19 patients. Therefore, calprotectin is demonstrably a foremost candidate biomarker for COVID-19 infections with both diagnostic and prognostic significance. Recent evidence suggests that calprotectin offers a potential therapeutic strategy in severe COVID-19 as well as other inflammatory diseases. Further studies should explore its role as a druggable target with several molecules such as the oral quinoline-3-carboxamide (e.g., tasquinimod), where it blocks the binding of calprotectin to TLR4 or RAGE (ACTRN12621000016831p). Such strategic therapeutic intervention could help decrease tissue damage, control pulmonary inflammation and thus halt the functional exhaustion seen in COVID-19. Other therapeutic strategies include lenzilumab (NCT04351152), a recombinant anti-human granulocyte-macrophage colony-stimulating factor (GM-CSF) antibody and preclinical antiCD33 monoclonal antibodies. Despite existing data, there is still a need for further functional studies on the heterodimer to facilitate a more detailed characterization of calprotectin [8,12].

## Figures and Tables

**Table 1:** Eligibility criteria.

PICOS	Inclusion	Exclusion
Participants	Adults ≥ 18 years with confirmed COVID-19 infection	Adolescents < 18 years
Intervention	Calprotectin level measured	Other diagnostic parameters used
Comparison	Severe and non-severe COVID-19 infections	-
Outcome	-Difference in calprotectin levels between the groups -Association between calprotectin level and clinical/laboratory assessment of disease activity -Association with risk of disease progress/therapeutic response	-
Study design	-Observational clinical studies -Randomized controlled trial (RCTs) -Case reports-Editorials	-Opinion papers, correspondents, review papers, healthcare guidelines, protocol -Non-human studies -Animal model and in-vitro studies

**Table 2:** Characteristics of included (quantitative) studies.

Author	Design (Follow-Up)	Sample Size (N) *	Country	Gender, % M **	Age, Mean Years (±SD) ***	% Deaths **	Study Quality ^b^	Peer-Review
	*Serum*							
Chen et al. (July, 2020) [14]	Retrospective	121 (ICU = 40, non-ICU = 81)	Wuhan (China)	64% (70 vs. 61)	63(ICU = 67, non-ICU = 62)	30% died (82.5% vs. 3.7%)	Good	Yes
De Guadiana R. et al. (August, 2020) [25]	Case series	66 (Non-survivors = 58, survivors = 8)	Cartagena (Spain)	65% (68 vs. 58)	Total = 61 ± 16: non-survivors = 74 ± 9, survivors = 60 ± 16	12% (i.e., 8) died	Good	Yes
Silvin et al. (August, 2020) [12]	Cohort	158 (severe = 50, non-severe = 39, healthy controls = 86)	France	44% (67 vs. 30)	53 (severe = 62, non-severe = 53, control = 50)	24% vs. 4%	Good	Yes
Shi et al. (July 2020) [26]	Cohort	172 (Room air group = 41; non-invasive oxygen = 71; invasive ventilation= 60)	Michigan (USA)	56%M	61.48 ± 17,7	NR	Good	Yes
Bauer et al. (November 2020) [27]	Cohort	19 (ICU = 8, non-ICU = 11)	Berlin (Germany)	42%M	67.6	10.5%	Good	Yes
	*Fecal*							
Effenberger et al., August 2020 [18]	Cohort	40 (COVID-19 with diarrhea = 22, without diarrhea = 18)	Innstruk Austria	Severe = 68%M, non-severe = 50%M	Severe = 72.3, non-severe = 58.4	NR	Moderate	Yes
Ojetti et al. (November 2020) [28]	Cohort	65 (19 vs. 46)	Rome (Italy)	77% (53 vs. 87)	38 (56 vs. 36)	NR	Good	Yes
Britton et al. (September, 2020) [29]	Retrospective	44 (31 vs. 13)	NY (USA)	48% (55 vs. 31)	56 (53 vs. 63)	16% (16 vs. 15)	Good	No

NB: ^b^ For the detailed appraisal scores using the quality assessment tools (Newcastle-Ottawa scale for cohort/case-control and National institute of Health for case-series) and process—see Tables S1 and S2 respectively inback matter [30,31,32]. * Data are expressed as frequency; ** overall % (%severe vs. %non-severe); *** Age, mean ± SD (severe vs. non-severe).

**Table 3:** The main results of quantitative studies that reported Calprotectin.

Author	Severe Group (Mean ± SD)	Non-Severe Group (Mean ± SD)	Confidence Interval (*p*-Value)	Primary Results/Conclusion
		*Serum*		
Chen et al. (June 2020) [14]	9220	7800	*p*-value = 0.0001	Calprotectin circulating level strongly correlated with mean oxygen score and is significantly raised in COVID-19 patients who died. Hence, a potential role in the assessment of prognosis in these patients. AUC for calprotectin (ICU vs. non-ICU) = 0.860, 85% sensitivity, 82.7% specific, 6195.015 cut-off (COVID-GRAM score 0.810, HMGB1—0.781). Serum calprotectin was highly correlated with quick-Sequential Organ Failure score (qSOFA) score and oxygen demand.
De Guadiana Romualdo et al. (August 2020) [25]	7900 (5060)	3540 (2270)	*p*-value < 0.001	Serum calprotectin correlated positively with ferritin, CRP, Calprotectin plasma level was significantly higher in non-survivors COVID-19 group, suggesting a possible prognostic value for serum calprotectin in COVID-19 infection.
Silvin et al. (August 2020) [12]	4983.4 (2815.1)	985.0 (1161.9)	*p*-value < 0.0001	Severe COVID-19 patients have a peripheral blood and lungs characterized by HLA-DRlow monocytes and immature neutrophils. They also possess higher calprotectin levels that correlates positively with neutrophil count and severity of COVID-19 infection. Absence of non-classical monocytes could select patients at high risk of ICU admission or death. Serum calprotectin correlated with the ROC AUC (discriminating capacity) of the plasma calprotectin level was 0.9590 (non-classical monocytes = 0.8705, CD16low = 0.7983, IFNα = 0.5613).
Shi et al. (July 2020) [26]	11251.26 (7776.52)	4709.79 (5214.13)	*p*-value < 0.0001	Calprotectin level was significantly raised in ICU group compared to the non-ICU group, which suggests that higher calprotectin levels are associated with higher deaths. Serum calprotectin also correlated with D-Dimer, qSOFA score (it reflects the functional state of the organs), COVID-GRAM risk scores. AUC for calprotectin (need invasive ventilation vs. No need for invasive ventilation) = 0.794 (CRP = 0.614, ferritin = 0.562).
Bauer et al. (November 2020) [27]	3770 (1765)	2080 (988)	*p*-value = 0.15	Calprotectin is a new and important discriminator in COVID-19 with regards to disease outcome especially multiple organ failure. Estimation of the serum levels of calprotectin can be easily adopted into routine laboratories and performs better than traditional biomarkers such as CRP, lactate and PCT (procalcitonin).
		*Fecal*		
Effenberger et al. (August 2020) [18]	80.2 (26.51)	17.3 (3.83)	*p*-value = 0.001	FC levels were significantly higher in COVID-19 patients with diarrhea and correlated positively with serum IL-6 but not with CRP and ferritin. Fecal SARS-CoV-2 was only detected in COVID-19 group with ongoing diarrhea but not in the other two groups.
Ojetti et al. (November 2020) [28]	71.3	11.9	*p*-value = 0.001	A significant association exist between high fecal calprotectin level and COVID-19 pneumonia as well as disease severity. Systemic involvement often accompanies the pneumonia even in asymptomatic COVID-19 patients. Such systemic involvements may not present with gastrointestinal symptoms but can be demonstrated by high fecal calprotectin level.
Britton et al. (September 2020) [29]	2.5	2.0	*p*-value = 0.12	SARS-CoV-2 RNA was seen in stools of 41% of patients, and is seen more in those that have diarrhea than in others who did not have diarrhea. Severe COVID-19 was associated with elevated IL-23 and intestinal virus-specific IgA level. Fecal calprotectin did not correlate with gastrointestinal symptoms or viral level detected.

NB: All data on calprotectin is in ng/mL, AUC Area under the curve, ROC Receiver operating characteristic, CLP calprotectin. Effenberger et al. [18]: Severe group = COVID-19 with diarrhea, non-severe group = COVID-19 without diarrhea. Silvin et al. [12], Severe group = critical patients, non-severe = mild/moderate patients.

**Table 4:** The main results of qualitative studies.

Author	Methodology	Primary Results/Conclusion
Unterman et al. (2020) [30]	Single cell analysis	Calprotectin circulating level is significantly elevated in COVID-19 patients who died. Hence, a potential role in the evaluation of prognosis in these patients.
Livanos et al. (2020) [31]	Single cell analysis	Study negates the concept of gut tropism. It reports that there is a significant decrease in severity and deaths of COVID-19 when patients present with GI symptoms such as diarrhea, nausea and vomiting

## Data Availability

Data supporting reported results can be found attached as an excel sheet file.

## Supplementary Materials

The following are available online at https://www.mdpi.com/2077-0383/10/4/775/s1, Figure S1: Meta-analysis (sensitivity analysis excluding three studies). Table S1: Newcastle-Ottawa Scale (NOS), Table S2: National Institute of Health (NIH) tool.
