# Supplementary material for: Calprotectin, an Emerging Biomarker of Interest in COVID-19: A Systematic Review and Meta-Analysis
Source: J Clin Med. 2021 Feb 15;10(4):775. doi: 10.3390/jcm10040775 (PMC7919496; doi:10.3390/jcm10040775)
Supplement: Supplementary file 1 [file jcm-10-00775-s001.pdf]

Appendix:

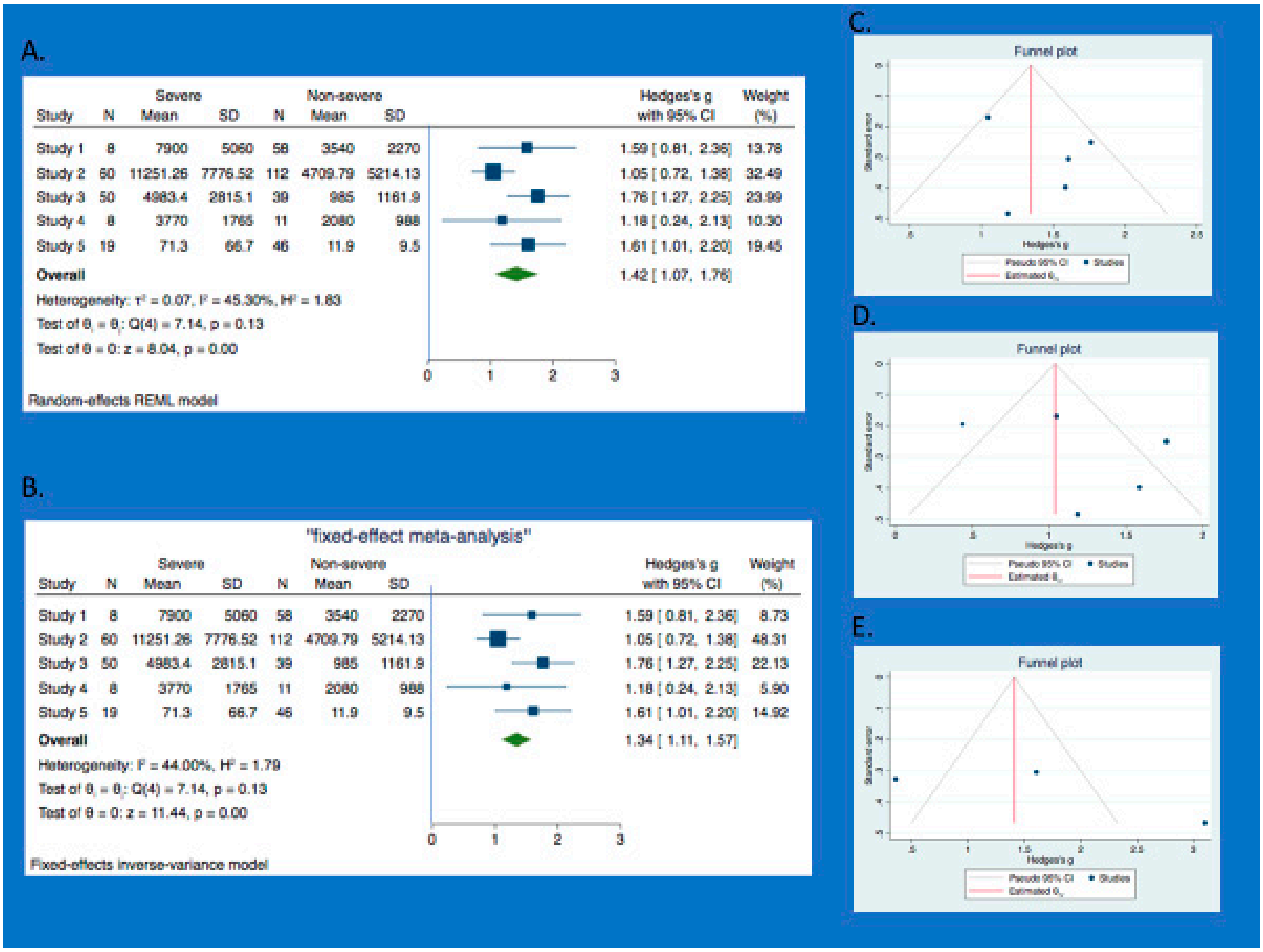

Figure S1: Meta-analysis (sensitivity analysis excluding three studies). A. Forest plot (REM) and B. Forest plot (FEM) comparing the mean differences in calprotectin level between severe and non-severe COVID-19. Studies 1-5 are respectively - De Guadiana et al [25]; Shi et al [26]; Silvin et al [12]; Bauer et al [27]; Ojetti et al [28]. C. Funnel plot (for the sensitivity analysis excluding three studies) shows no publication bias, an improvement from the total cohort funnel plot shown in figure 3c. D. Funnel plot (Subgroup analysis - Serum group) shows no publication bias. E. Funnel plot (Subgroup analysis - faecal group) shows some evidence of publication bias with much asymmetry.

Table S1: Quality assessment for the included cohort / case-control studies using Newcastle-Ottawa Scale[31-32]

Study	**Item 1**	**Item 2**	**Item 3**	**Item 4**	**Item 5**	**Item 6**	**Item 7**	**Item 8**	**Score**
Chen et al[14]	*	*	*	*	* *	*	*	*	**9/9**
Shi et al[26]	*	*	*	*	*	*	*	*	**8/9**
Silvin et al[12]	*	*	*	*	**	*	*	*	**9/9**
Bauer et al[27]	*	*	*	*	*	*	*	*	**8/9**
Effenberger et al[18]	*	*	*	*	*	*	*	**NR**	**7/9**
Ojetti et al[28]	*	*	*	*	*	*	*	*	**8/9**
Britton et al[29]	*	*	*	*	*	*	*	*	**8/9**
Unterman et al[33]	*	*	*	*	*	*	*	*	**8/9**
Livanos et al[34]	*	*	-	-	-	*	*	**NR**	**4/9**

NB: Items were as follows for cohort studies: 1-representativeness of the exposed cohort; 2-selection of the nonexposed cohort; 3-ascertainment of exposure; 4-demonstration that the outcome of interest was not present at the start of the study; ****5**-a comparability of cohorts on the basis of the design or analysis; 6-assessment of the outcome 7-follow-up period was long enough for outcomes to occur; 8-adequacy of follow-up evaluation (>75% follow-up evaluation, or description for those lost).

Items were as follows for case-control studies: 1-Is the case definition adequate 2-representativeness of the cases; 3-selection of controls; 4-definition of controls; 5-comparability of cases and controls on the basis of the design or analysis; 6-ascertainment of exposure; 7-same method of ascertainment for cases and controls; 8-non-response rate. **Item 5 for any of the study designs is scored double stars while the rest of the items are scored 1 star each.

Table S2: Quality assessment for included case series studies using NIH tool[30]

Criteria	De Guadiana et al, 2020[25]<br>Yes/No
1. Was the study question or objective clearly stated?	Yes
2. Was the study population clearly and fully described, including a case definition?	Yes
3. Were the cases consecutive?	Yes
4. Were the subjects comparable?	Yes
5. Was the intervention clearly described?	Yes
6. Were the outcome measures clearly defined, valid, reliable, and implemented consistently across all study participants?	Yes
7. Was the length of follow-up adequate?	Yes
8. Were the statistical methods well-described?	Yes
9. Were the results well-described?	Yes
Quality rating	Good

NB: NIH Quality assessment tool for case series studies. CD, cannot determine; NA, not applicable; NR, not reported
